# Supplementary material for: “Self-Assisted” Amoeboid Navigation in Complex Environments
Source: PLoS One. 2011 Aug 4;6(8):e21955. doi: 10.1371/journal.pone.0021955 (PMC3150345; doi:10.1371/journal.pone.0021955)
Supplement: Table S1 — Parameter values. (DOCX) [file pone.0021955.s001.docx]

**Supporting Table S1**

| C_1_ | 2.5 |
| --- | --- |
| C_p_ | 0.128 |
| γ1 | 6.5 |
| γ2 | 3.2 |
| γ3 | 0.9 |
|  | 0.01 |
| λ | 0.025 |
| dt | 0.001 |
| Patch lifetime | 1.2-1.6 minutes |
| Patch size | 25-40 μm |
