# Supplementary material for: “Self-Assisted” Amoeboid Navigation in Complex Environments
Source: PLoS One. 2011 Aug 4;6(8):e21955. doi: 10.1371/journal.pone.0021955 (PMC3150345; doi:10.1371/journal.pone.0021955)
Supplement: Text S1 — Amoeboid motion: model description and computational details. (DOCX) [file pone.0021955.s002.docx]

**Supporting Text S1**

As described in the main text, the model cell is represented by connected nodes. Typically, 100-250 nodes are used to represent a single cell. The nodes are conveniently stored as a double-linked chain.

In this work, we do not explicitly concern ourselves with modeling the gradient sensing mechanism that detects the external chemical concentration field. How a cell determines its front has been the subject of many theoretical studies [[52](#_ENREF_52), [53](#_ENREF_53)]. Here, we directly assume that the cell’s front is determined through the formation of an internal compass, which is simply determined by the external gradient direction. Specifically, we choose the internal compass direction, *ϕ_int_*, to be the external direction *ϕ_ext_* plus some added noise:

*ϕ_int=_ϕ_ext+_η_ϕ_* (3)

The term *η_ϕ_* represents all the possible fluctuations in the directional sensing process and is drawn from a Gaussian distribution with zero mean and width σ. We assume that the width of the noise distribution is inversely proportional to the steepness of the gradient such that the directional sensing process is more accurate for steeper gradients. σ is therefore dynamically varied according to the local difference of the chemoattractant between the cell’s front and back (see also the main text). The front of the cell is then chosen to be the point on the membrane (i.e. linked chain of nodes) that is closest to the direction of the internal gradient .

The total force acting on each node is:

$F_{tot}=f_{p}(a)-\gamma(\kappa-\kappa_{0})-C_{1}(A-A_{0})-\lambda v$ (A1)

In this equation, the first term is the membrane protruding force, which couples the signaling and the motility. In this simulation, the signaling system is represented by the activation patch, and the protruding force is simply a linear function of the activation level. This approach is based on experimental observations, showing that localization of RasGTP to the membrane of chemotaxing *Dictyostelium* cells occurs in the form of finite-time patches, which strongly correlate with pseudopod emergence. In the simulation, the center of the patch is located in the node that best corresponds to the internal compass direction. The lifetime of a patch is drawn from a uniform distribution in the range 1-2 minutes (for simplicity; a Gaussian distribution can also be taken), according to the experimental data. Once a patch’s lifetime is over, the patch is degraded and a new patch is similarly created. The activation patch width *W* is between 25-40 μm, approximately, and its magnitude *a* is with *d* being the distance from the center of the patch. The force is simply and is a constant (see list of parameter values in Supporting Table T1).

The second term in the right-hand side of Eq. (A1) describes the cortical tension, which depends on the local curvature *κ*. represents the membrane rigidity, with higher values of corresponding to more rigid membranes. *κ_0_* is the spontaneous curvature of the cell, which is the equilibrium curvature when the total force is zero, namely for a circular cell of radius R. We choose the back part of the cell, defined as the portions of the membrane for which *a=0*, to have a cortical tension (*γ_1_*) that is about twice as high as the cortical tension (*γ_2_*) in the front part of the cell where *a>0*. This assumption is based on experimental data of *Dictyostelium discoideum*, showing Myosin-II accumulation at the back of cell, resulting in higher cell rigidity compared to the cell front[[54](#_ENREF_54), [55](#_ENREF_55)]. In addition, we have empirically discovered that in order to produce cell extensions with large aspect ratio, i.e. long and narrow, and to get the “valleys” between them to have a reasonable shape, we need to allow regions of the membrane with negative curvature to have a value *γ_3_* that is smaller yet. A possible origin of this effect lies in that we are using a two-dimensional model to describe a three-dimensional cell (albeit moving within a limited three dimensional space). The tension force in 3d should of course be proportional to the total curvature and it might be the case that negative in-plane curvature tends to cancel the positive out-of-plane curvature, resulting in small net effect. In [[30](#_ENREF_30)] we show the role of this effect and how it influences the cell shape.

The third term ensures that the cellular area *A* (which is the equivalent of the cellular volume in the 3D case) remains constant and can be viewed as an effective pressure. Finally, the last term represents an effective drag force, proportional to the local velocity *v*, and determines the time a protrusion continues to move after the protruding force has vanished. This term also yields a limit on the maximal speed, so that a constant force in one direction results in a constant speed rather than an unrealistic constant acceleration. The evolution of each node is found by solving:

$\frac{dv}{dt}=F_{tot}$ **(A2)**

The entire simulation is performed in the following sequential steps: First, the force on each node is computed using Eq. (A1). Then, the velocity of each node is calculated and the nodes are advanced simultaneously according to Eq. (A2). The time scale in the simulations can be converted to physical units by comparing the simulation cell speed to the cell speed obtained in the experiments and by taking a cell length that is comparable to the experimental dimensions of a cell. A complete list of parameter values is given in Supporting Table T1.

The total number of membrane nodes is not constant and nodes are added and removed to keep the distance between them within a given range. When the membrane is extended, nodes are added at the tip where the membrane “stretches” and removed at the back of the cell. Care was taken such that the total activation level (equivalent to the overall amount of a membrane-bound chemical) remained constant during this reparametrization. The list of node locations was recorded every 25000 iterations and used to construct the cell contour and the cell body. The cell was drawn using Matlab and the separate frames were constructed into a movie.
